# Unraveling the transcriptomic landscape of brain vascular cells in dementia: A systematic review

**DOI:** 10.1002/alz.14512

**Published:** 2025-01-14

**Authors:** Michael Sewell, Nela Fialova, Axel Montagne

**Affiliations:** ^1^ UK Dementia Research Institute at the University of Edinburgh Edinburgh UK; ^2^ British Heart Foundation ‐ UK Dementia Research Institute Centre for Vascular Dementia Research at the University of Edinburgh Edinburgh UK; ^3^ Centre for Clinical Brain Sciences University of Edinburgh Edinburgh UK

**Keywords:** Alzheimer's disease, cerebral small vessel disease, endothelial cell, pericyte, transcriptomics

## Abstract

**INTRODUCTION:**

Cerebrovascular dysfunction plays a critical role in the pathogenesis of dementia and related neurodegenerative disorders. Recent omics‐driven research has revealed associations between vascular abnormalities and transcriptomic alterations in brain vascular cells, particularly endothelial cells (ECs) and pericytes (PCs). However, the impact of these molecular changes on dementia remains unclear.

**METHODS:**

We conducted a comparative analysis of gene expression in ECs and PCs across neurodegenerative conditions, including Alzheimer's disease (AD), Huntington's disease, and arteriovenous malformation, utilizing transcriptomic data from published postmortem human tissue studies.

**RESULTS:**

We identified differentially expressed genes (DEGs) consistently dysregulated in ECs and PCs across these pathologies. Notably, several DEGs are linked to vascular cell zonation and genetic risks for AD and cerebral small vessel disease.

**DISCUSSION:**

Our findings provide insights into the cellular and molecular mechanisms underlying vascular dysfunction in dementia, highlight the knowledge gaps, and suggest potential novel vascular therapeutic targets, including genes not previously investigated in this context.

**Highlights:**

- Systematic review of differentially expressed genes (DEGs) in vascular cells from neurodegenerative single‐nuclear RNA‐sequencing (snRNA‐seq) studies.
- Identify overlapping DEGs in multiple vascular cell types across studies.
- Examine functional relevance and associations with genetic risk for common DEGs.
- Outline future directions for the vascular omics field.

## 1 BACKGROUND

The brain vasculature plays pivotal roles in both the maintenance of brain health and in various central nervous system disorders, notably several dementia and neurodegenerative disorders such as Alzheimer's disease (AD).^1^, ^2^, ^3^ A compelling body of evidence has demonstrated that vascular dysfunction arises early in the onset and progression of these diseases,^4^, ^5^, ^6^ and is strongly associated with symptoms including cognitive decline,^6^, ^7^ as well as key pathological hallmarks such as amyloid beta (Aβ) plaques.^8^ Targeting vascular abnormalities therefore represents a promising therapeutic avenue for these disorders. However, the development of vascular‐focused treatments is currently hindered by our limited understanding of the cellular and molecular mechanisms underlying vascular dysfunction in these diseases.

Nonetheless, significant progress has been made in the field in recent years with the advent of omics technologies that enable profiling of the brain vascular cells at a single‐cell resolution, capturing both the whole transcriptome and heterogeneity of vascular cells such as endothelial cells (ECs) and pericytes (PCs).^9^, ^10^ Indeed, several studies utilizing single‐nuclear RNA‐sequencing (snRNA‐seq) of vascular cells from postmortem tissues of patients with dementia and various neurodegenerative disorders have demonstrated that vascular dysfunction is closely linked to extensive transcriptomic alterations throughout the brain vasculature, including in AD,^11^, ^12^, ^13^, ^14^ Huntington's disease (HD),^15^ and arteriovenous malformations (AVMs).^16^ However, the importance of these transcriptomic changes to disease etiology remains unknown, although recent work has implicated associations with genetic risk, specifically in AD.^11^, ^13^

Continued expansion of this body of literature will help build a consensus as to which genes and pathways are most relevant to disease pathology and thus pinpoint possible vascular therapeutic targets. In light of numerous omics studies concerning the brain vasculature published in recent years, we took this opportunity to systematically review and synthesize findings across these studies to identify common differentially expressed genes (DEGs) in both vascular cell types and subtypes in neurodegenerative diseases. Our analysis has identified a multitude of overlapping DEGs in the brain vasculature across six recent snRNA‐seq studies. For DEGs of interest, we discuss their potential associated functions and relevant roles in disease progression, including in the case of AD, for which DEGs may be associated with genetic risk. Finally, we discuss future directions for the vascular omics field to further enrich our understanding of the role of vascular transcriptomic changes in these diseases.

## 2 METHODS

### 2.1 Search strategy and selection criteria

We reviewed the available literature to identify topics related to transcriptomic changes of the brain human vasculature in various neurological diseases. The search was performed on Medline via Ovid, covering publications from April 2019 up to April 2024. To obtain the most comprehensive results possible, we combined headings and search terms related to brain vasculature with those specific to single‐cell and single‐nuclei RNA sequencing. We then combined this search with neurodegeneration and brain disorders prior to limiting articles relating to humans published in the last 5 years. The full search strategy (Table S1A,B) resulted in 34 publications, which were then manually checked alongside with reference lists in reviews and original articles for additional relevant publications. All identified articles were inspected against the previously determined inclusion and exclusion criteria. The inclusion criteria were defined as follows: snRNA‐seq, cells of the vasculature (ECs/PCs), neurological diseases, human, last 5 years. The exclusion criteria were defined as: rodents, review articles, abstracts, and studies with no original data set. In addition, we selected studies for analysis that had profiled significantly high numbers of vascular nuclei in their data sets (>10,000), as these studies are likely the most sufficiently powered to detect vascular DEGs in postmortem tissue.

**RESEARCH IN CONTEXT**

1. **Systematic review**: We conducted a comparative analysis of transcriptomic alterations in brain vascular cells, specifically endothelial cells (ECs) and pericytes (PCs), across neurodegenerative conditions such as Alzheimer's disease (AD). Utilizing databases such as Medline, we identified and evaluated transcriptomic studies to compile differentially expressed genes (DEGs) and assess their relevance to cerebrovascular dysfunction in dementia.
2. **Interpretation**: Our findings reveal a consistent set of DEGs dysregulated in ECs and PCs, some of which are linked to genetic risks associated with AD and cerebral small vessel disease. This work enhances our understanding of the molecular mechanisms driving vascular dysfunction in dementia, indicating a crucial role for vascular health in neurodegenerative processes.
3. **Future directions**: Future research should focus on (1) elucidating the functional roles of identified DEGs in vascular pathology; (2) investigating the interplay between vascular and neuronal changes in dementia; and (3) exploring novel therapeutic targets derived from vascular transcriptomic profiles to address cerebrovascular contributions to neurodegeneration.

### 2.2 Data analysis

Genes that had been defined as DEGs for ECs and PCs by the authors were extracted from studies of interest. List of genes implicated in AD/cerebral small vessel disease (SVD) genetic risk were taken from references.^13^, ^17^, ^18^ Overlap between studies was then determined in R (v.4.3.2), with the “ggvenn” and “UpSetR” packages employed for data visualization and figure generation. Gene ontology (GO)–enrichment analysis for functional annotation of overlapping DEGs was performed using the “clusterProfiler”^19^ package. For protein–protein interaction (PPI) network analysis, lists of overlapping DEGs were inputted into STRING,^20^ which based on known functional and physical interactions between genes subsequently generated networks. We then filtered out interactions with a STRING confidence score of less than 0.7 (high confidence interval [CI]) in line with previous work,^21^ and imported networks into Cytoscape^22^ for visualization.

## 3 RESULTS

### 3.1 Overlapping EC DEGs across neurodegenerative conditions and associated pathologies

Previously published snRNA‐seq studies have focused particularly on profiling transcriptomes of ECs, which are thought to play significant roles in the pathophysiology of several neurodegenerative disorders such as AD and HD.^4^ We, therefore, sought to investigate whether there were any common EC DEGs in disorders and pathologies that have been investigated previously through snRNA‐seq on postmortem tissue from human cases, including those with an AD^13^, ^14^ or HD^15^ diagnosis, as well as those with AVMs, a pathology that is also frequently reported in diseases such as AD.^16^

**FIGURE 1:**
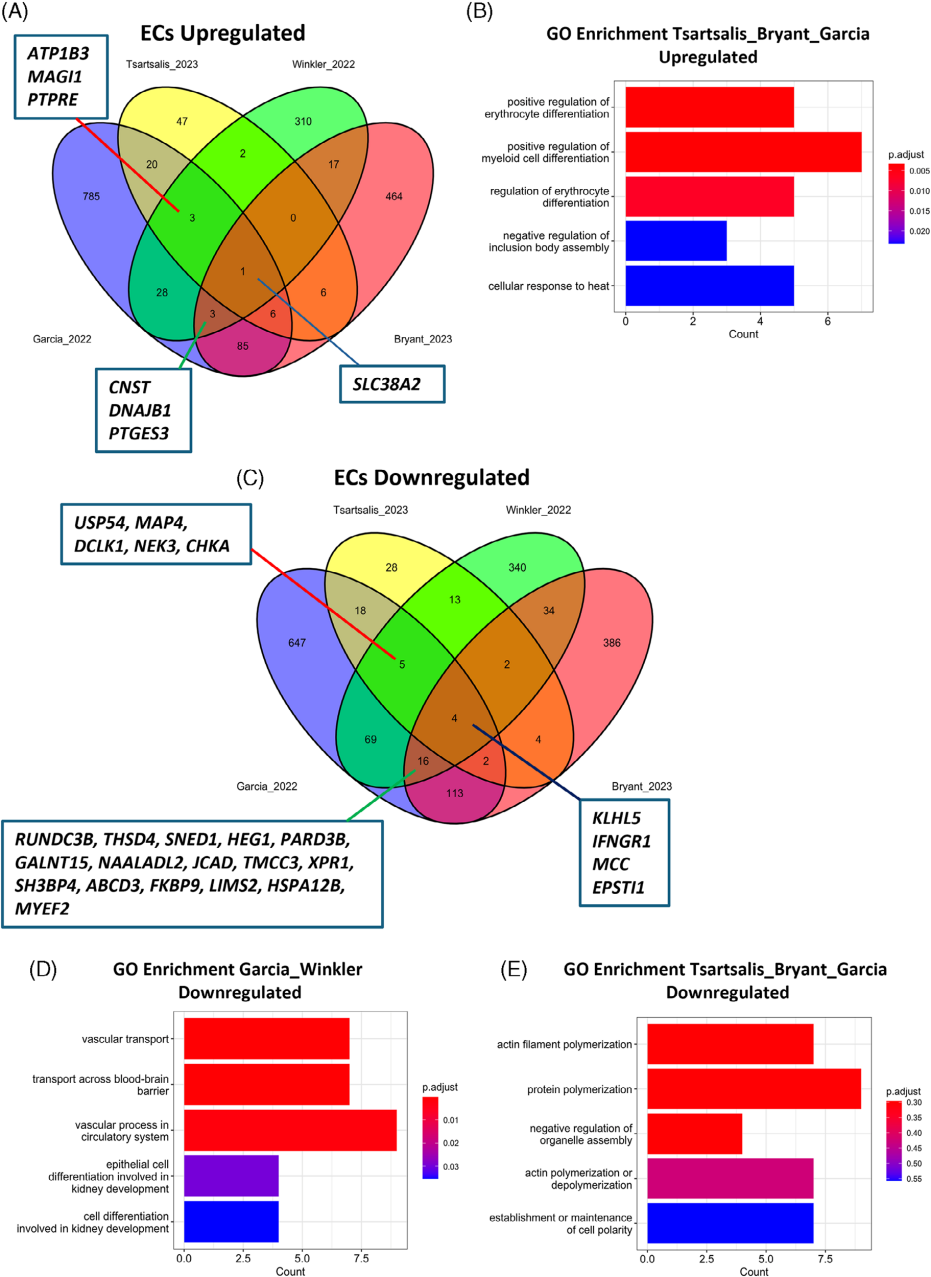
Shared endothelial cell DEGs across AD (Tsartsalis et al., Bryant et al.), HD (Garcia et al.), and AVM (Winkler et al.) postmortem tissue studies. (A) Venn diagram showing overlapping upregulated EC DEGs across studies. (B) Bar plot from GO‐enrichment analysis on shared DEGs identified in Garcia et al. study and either Bryant et al. or Tsartsalis et al. studies, showing top five enriched terms. (C) Venn diagram showing overlapping downregulated EC DEGs across same studies in (A). (D, E) Bar plots from GO‐enrichment analysis showing top five associated terms on shared genes identified by Garcia et al. with Winkler et al. (D) and either Bryant et al. or Tsartsalis et al. (E). AD, Alzheimer's disease; AVM, arteriovenous malformation; DEGs, differentially expressed genes; EC, endothelial cell; GO, Gene ontology; HD, Huntington's disease.

We noted a considerable amount of overlap of upregulated EC DEGs between AD and HD studies, with 95 shared DEGs between the studies of Bryant et al. (AD) and Garcia et al. (HD) (Figure 1A). In addition, 30 genes were shared between the studies of Tsartsalis et al. (AD) and Garcia et al. (Figure 1A). GO‐enrichment analysis revealed that the most significantly associated terms with the 118 overlapping AD/HD genes (Table S2A) included “positive regulation of erythrocyte differentiation,” “positive regulation of myeloid cell differentiation,” and “cellular response to heat” (Figure 1B). Indeed, several of these shared upregulated genes are members of the heat shock protein (HSP) family, including *HSPA1B, HSP90AA1, DNAJB1, HSPB1, HSP90AB1*, and *DNAJB6*. The corresponding proteins are thought to be critical for cellular proteostasis maintenance and protection against stress,^23^ which may be upregulated in response to Aβ, tau, and mutant huntingtin (mHTT) aggregation in these diseases. PPI network analysis also revealed that these heat‐shock proteins interact with proteins encoded by other DEGs that have been implicated previously in AD and HD etiology (Figure S1). Notably, *HSP90AA1*, *HSP90AB1*, and *HSP1B* all interact with the neuroinflammatory‐associated transcription factor signal transducer and activator of transcription 3 (*STAT3*), the inhibition of which in ECs has been shown previously to attenuate amyloid pathology and cognitive deficits in an AD mouse model.^24^ Heat shock proteins also interact with the hypoxia‐associated transcription factor hypoxia‐inducible factor 1‐alpha (*HIF1A*),^23^ the increased expression of which along with another hypoxia‐associated DEG and transcription factor forkhead box O3 (*FOXO3*) may arise from pathology such as reduced cerebral blood flow in AD and HD patients.^25^, ^26^ Although this may be neuroprotective by stimulating processes such as angiogenesis in response to hypoxia, increased expression of these factors may also play a detrimental role in disease progression, with previous work showing that increased *HIF1A* expression perturbs microglial metabolism and exacerbates amyloid pathology in AD mice.^27^

More limited overlap was observed between AD and HD upregulated EC DEGs with those identified in postmortem tissue with AVMs (Figure 1A, Table S2B). Indeed, only seven common upregulated DEGs: *SLC38A2*, *CNST, DNAJB1, PTGES3*, *ATP1B3, MAGI1*, and *PTPRE* were observed in postmortem tissue from all three diseases (Figure 1A). Conversely, significantly more overlap was noted for downregulated DEGs between AVMs and HD, with 94 shared genes between the studies of Garcia et al. and Winkler et al., four of which (*KLHL5, IFNGR1, MCC*, and *EPSTI1*) were also downregulated in both AD studies (Figure 1C, Table S2C). GO‐enrichment analysis showed that the most significant associated terms for these overlapping genes included “vascular transport” and “transport across the blood‐brain‐barrier” (Figure 1D). Both of these terms are related to the downregulation of genes encoding transporters at the blood–brain barrier (BBB):  ATP synthase–binding cassette transporters (ABC) *ABCB1* and *ABCG2*, major facilitator superfamily domain‐containing protein 2 (*MFSD2A*), and solute carrier family members *SLC16A1*, and *SLC38A5*,^28^, ^29^ as well as transferrin receptor (*TRFC*), which is involved in regulating iron uptake and homeostasis.^30^ A considerable amount of overlap was also found between AD and HD studies, with 158 identified shared genes between either Tsartsalis et al. or Bryant et al. AD studies, and the Garcia et al. HD study (Figure 1C, Table S2D), with the highest number of genes associated with GO‐enrichment terms “actin filament polymerisation” and “protein polymerisation” (Figure 1E). These GO terms may possibly pertain to altered cell adhesion properties in response to Aβ and/or tau aggregates, as has recently been shown to occur in capillary ECs through the ROCK/RhoA pathway, for which components such as Rac family small GTPase 1 (*RAC1*)^32^ was a detected downregulated DEG. Components of the transforming growth factor‐beta (TGF‐β)‐SMAD signaling pathway such as transforming growth factor beta receptor 2 (*TGFBR2*) and SMAD family member 6 (*SMAD6*) were also downregulated, which may also pertain to altered cell adhesion given the known roles of this signaling cascade in actin‐mediated cytoskeleton remodelling.^33^

### 3.2 Are EC DEGs associated with specific arteriovenous subsets?

Previous work has demonstrated the presence of distinct EC subsets along the arteriovenous axis, which present unique transcriptomic profiles.^10^ More recently, several snRNA‐seq studies have also identified DEGs in these specific subsets in AD postmortem tissue.^11^, ^12^ We, therefore, sought to compare DEGs of AD studies that considered all ECs as a single population^13^, ^14^ with studies that had captured arteriovenous subset DEGs,^11^, ^12^ to see whether any DEGs are associated with a particular zonal subpopulation.

**FIGURE 2:**
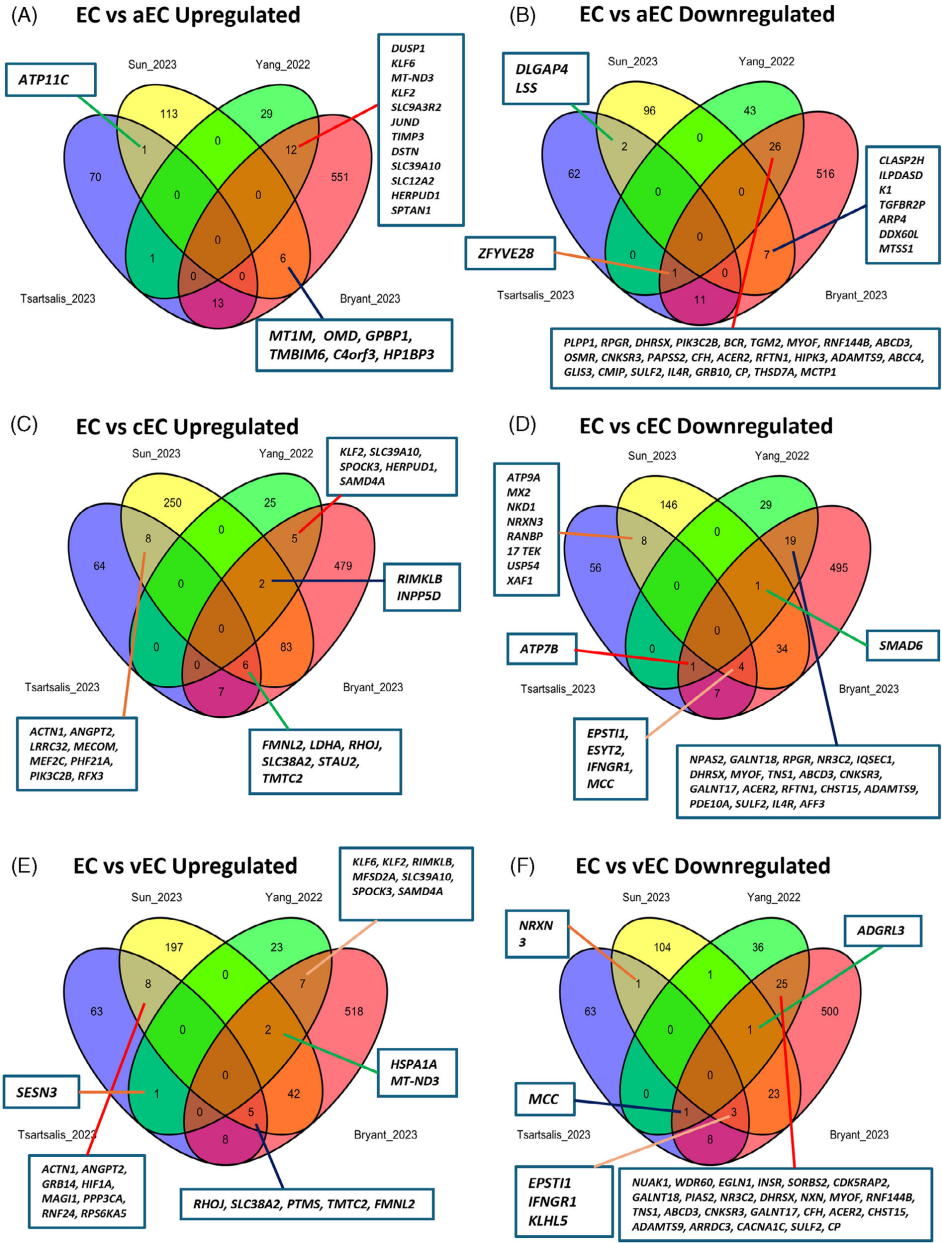
Comparison of universal versus arteriovenous subset endothelial cell upregulated and downregulated DEGs across AD studies. Venn diagrams showing overlap between DEGs from universal EC populations (Bryant et al. and Tsartsalis et al.) with specific arteriovenous subsets identified by Sun et al. and Yang et al.: aECs (A, B), cECs (C, D), and vECs (E, F). AD, Alzheimer's disease; aECs, arterial ECs; cECs, capillary ECs; DEGs, differentially expressed genes; EC, endothelial cell; vECs, venous ECs.

Of interest, we found limited evidence for DEGs, which were universally altered across all arteriovenous subsets: arterial ECs (aECs), capillary ECs (cECs), and venous ECs (vECs). Indeed, only seven DEGs (upregulated: *KLF2* and *SLC39A10*; downregulated: *DHRSX*, *MYOF*, *ABCD3*, and *ADAMTS9*) identified in the Bryant *et al.* EC population were found to be differentially expressed in the aEC, cEC, and vEC subsets of the Yang et al. study, with none from the Tsartsalis et al. study found to be altered in all subsets from either the Yang et al. or Sun et al. studies (Figure 2). Moreover, several DEGs of interest common to multiple neuropathologies also appear to be associated with a particular arteriovenous subset. For example, hypoxia‐associated transcription factor *HIF1A* and BBB transporter *MFSD2A* were found to be upregulated specifically in the vEC subset (Figure 2E), whereas *INPP5D*, a gene found upregulated in both AD and HD studies (Table S2A), was found to be altered specifically in cECs of the Yang et al. study (Figure 2C). TGF‐β‐SMAD signaling–associated genes *TGFBR2* and *SMAD6* were also found to be downregulated only in aEC and cEC subsets, respectively (Figure 2B,D). A large proportion of genes for subsets of the Yang et al. and Sun et al. studies were also not identified as DEGs in the Bryant et al. and Tsartsalis et al. studies, notably for aEC subsets (Figure 2A,B). Together, these findings highlight the importance of considering DEGs in individual EC subsets, which may be overlooked if all ECs are solely analyzed as one population. It should be noted, however, that there was very limited overlap observed across subsets between the Yang et al. and Sun et al. studies themselves, which possibly reflects differences in cell isolation techniques, with Yang et al. specifically performing vascular‐enrichment steps prior to sequencing.

### 3.3 Overlapping PC DEGs across neurodegenerative conditions and associated pathologies

**FIGURE 3:**
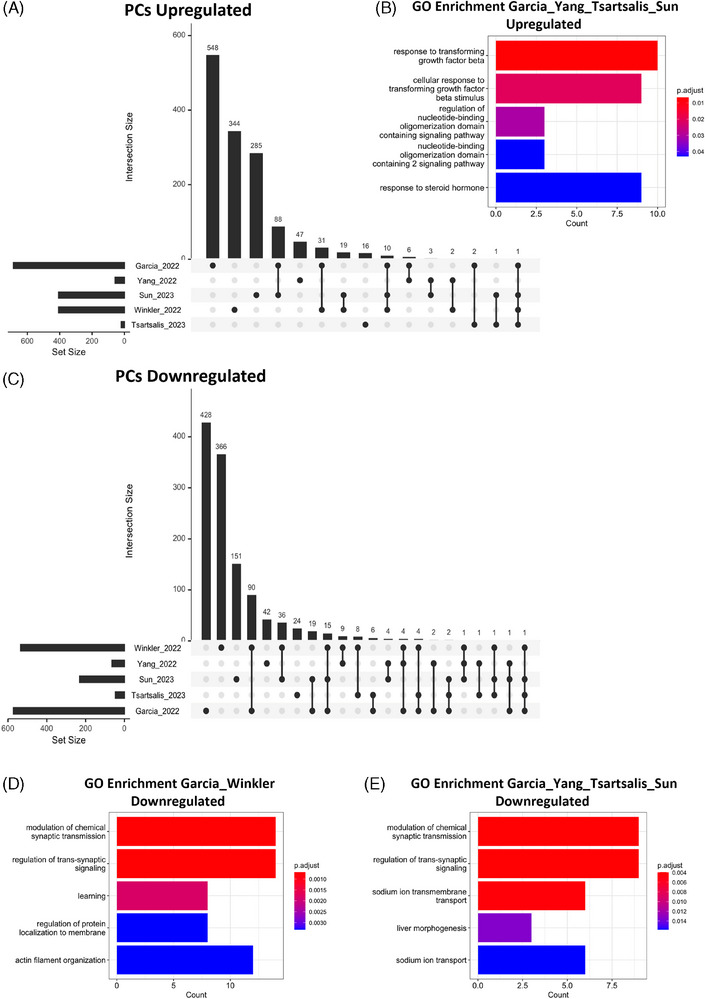
Common DEGs across pericyte populations in neurodegenerative diseases. (A) Upset plot showing overlapping upregulated genes between AD (Sun et al./Yang et al./Tsartsalis et al. studies), HD (Garcia et al.) and AVM (Winkler et al.) studies. (**B**) Bar plot showing top five associated terms from GO‐enrichment analysis on genes identified in Garcia et al. and one of Sun et al./Yang et al./Tsartsalis et al. (C) Upset plot showing downregulated gene overlap across the same studies in A. (D, E) Bar plots showing top five GO‐terms from GO‐enrichment analysis for shared genes between Garcia et al. and Winkler et al. (D) and one of Sun et al./Yang et al./Tsartsalis et al. studies (E). AD, Alzheimer's disease; AVM, arteriovenous malformation; DEGs, differentially expressed genes; GO, Gene ontology; HD, Huntington's disease.

Having assessed the overlap between EC DEGs, we then turned our attention to examining potential shared DEGs across studies for PC populations (Figure 3), another vascular cell type that closely interacts with ECs, and which themselves are being implicated increasingly in the pathogenesis of neurodegenerative diseases.^34^ As for ECs, we found a large amount of overlap of PC DEGs between AD and HD studies, with 107 upregulated DEGs shared between the Sun et al., Yang et al., or the Tsartsalis et al. AD and Garcia et al. HD studies (Figure 3A, Table S3A). GO‐enrichment analysis revealed that these shared upregulated genes were most significantly associated with terms such as “response to transforming growth factor beta (TGF‐β)” and “cellular response to transforming growth factor stimulus” (Figure 3B). These terms pertain to the upregulation of overlapping genes such as transforming growth factor beta receptor 1 (*TGFB1*) and its interactor latent transforming growth factor beta binding protein 1 (*LTBP1*), as shown in the PPI network analysis for these genes (Figure S2A). In AD, genes such as *TGFB1* have been studied extensively in the context of microglia, with its encoding cytokine TGF‐β1 thought to promote the adoption of microglial anti‐inflammatory phenotypes.^35^ Such a concept may also be applicable to PCs, which are thought to mediate the neuroinflammatory response in tandem with other brain cells such as microglia.^36^ Indeed, previous work has shown that TGF‐β1 can polarize PCs toward anti‐inflammatory phenotypes in vitro,^37^ which may similarly occur in AD to attenuate the neuroinflammatory response in late‐stage disease.

However, it is equally plausible that upregulation of many of these genes may be detrimental to disease pathogenesis. As for ECs, we also saw upregulation of certain heat shock protein genes, such as *HSPA1A* (Table S3A), which in the cerebrospinal fluid have been associated previously with cognitive impairment in AD.^38^ Another gene that was upregulated in AD and HD PCs that is also associated with cognitive impairment was the gene encoding epidermal growth factor receptor (*EGFR*), an interacting partner of many other detected DEGs (Figure S2A). Upregulation of *EGFR* has been implicated previously in Aβ‐mediated neurotoxicity in AD,^39^ as well as in promoting reactive astrogliosis and release of pro‐inflammatory cytokines.^40^ However, whether PC‐specific *EGFR* upregulation may play a role in driving pathology has yet to be addressed.

Our meta‐analysis also identified several downregulated genes in PCs across studies that have been associated previously with neurodegenerative disease pathology overlapping across studies (Figure 3C). This included the gene encoding platelet‐derived growth factor receptor β (*PDGFRB*), the altered expression of which has heavily been implicated in BBB breakdown in AD,^41^, ^42^ and which was downregulated in the studies of Garcia et al., Winkler et al., and Sun et al. (Table S3B,C). Similarly, downregulation was observed in the same studies of its interacting partner *FGF1* (Figure S2B,C), which has also been implicated in the pathogenesis of AD.^43^ Moreover, there was a considerable amount of overlap of PC downregulated genes with 114 shared genes between the Winkler et al. and Garcia et al. studies (Figure 3C, Table S3B), as well as 54 shared genes between the Garcia et al. and one of the examined AD studies (Figure 3C, Table S3C). GO‐enrichment analysis showed that both shared gene sets are significantly associated with terms such as “modulation of chemical synaptic transmission” and “regulation of trans‐synaptic signaling,” which are related to genes such as carbonic anhydrase (*CA1*) and *SLC12A2* (Figure 3D,E). PCs have been shown previously to modulate synaptic neurotransmission in the early stages of inflammatory responses to acute infection,^44^ which may be perturbed in these disease contexts given the known importance of neuroinflammation in each of these pathologies.^45^, ^46^, ^47^

### 3.4 Are vascular DEGs associated with genetic risk in AD?

Although the presence of a large number of vascular cell DEGs in neurodegenerative disease postmortem tissue has been ascertained, whether altered expression of these genes plays a causal role in disease pathology is not clear. Potential causality for vascular cell gene‐expression changes in AD has been implicated in two recent snRNA‐seq studies that performed sequencing on vascular‐enriched preparations. Both of these studies showed the enrichment of expression of AD risk genes in vascular cells, in addition to microglia,^11^, ^13^ suggesting roles for these cells and their transcriptomic signatures in the genetic pathophysiology of AD. We, therefore, sought to investigate whether any of these risk genes are also considered as DEGs in vascular cells across AD studies.

**FIGURE 4:**
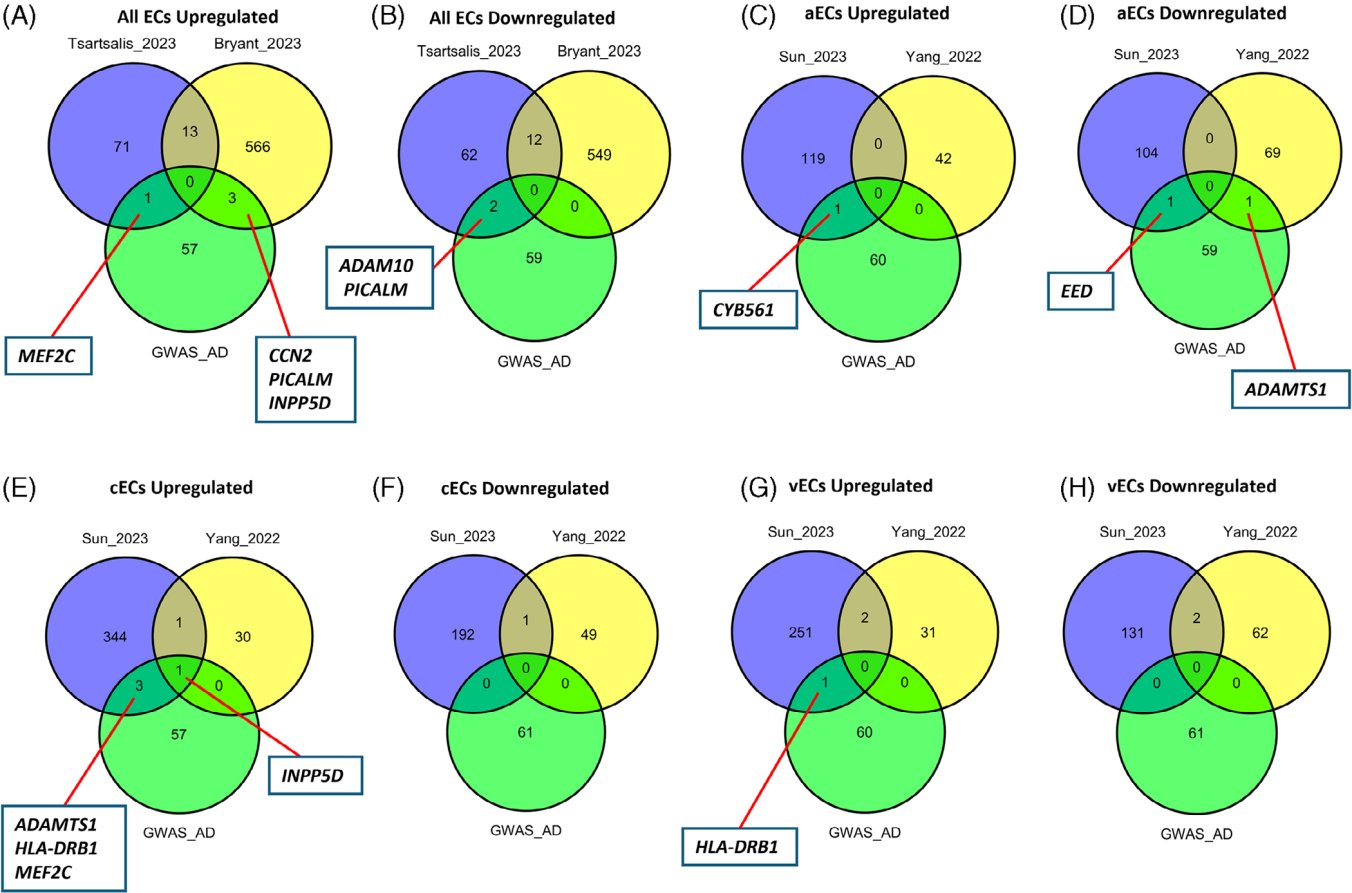
Comparison of AD studies’ EC DEGs with AD GWAS risk genes (obtained from Tsartsalis et al.). (A, B) Comparison for universal EC populations for aECs (C, D), for cECs (E, F), and for vECs (G, H). GWAS gene list obtained from^13^. AD, Alzheimer's disease; aECs, arterial ECs; cECs, capillary ECs; DEGs, differentially expressed genes; EC, endothelial cell; GWAS, genome‐wide association stuy; vECs, venous ECs.

We first examined whether any AD genome‐wide association studies (GWAS) risk genes are differentially expressed in EC populations (Figure 4), both in universal populations (Figure 4A,B) and subsets including aECs (Figure 4C,D), cECs (Figure 4E,F), and vECs (Figure 4G,H). Both Tsartsalis et al. and Bryant et al. detected altered expression of phosphatidylinositol‐binding clathrin assembly protein (*PICALM*) (Figure 4A,B) across all ECs. *PICALM* is a gene thought to be central to endocytosis, autophagy, and cholesterol and iron homeostasis, and has been implicated in a neuroprotective context in both amyloid and tau pathology.^48^, ^49^ Tsartsalis et al. also identified myocyte‐specific enhancer factor 2C (*MEF2C*) as an upregulated DEG (Figure 4A), as did Sun et al. specifically in cECs (Figure 4E). *MEF2C* encodes a transcription factor that has been shown previously to modulate microglial inflammatory response,^50^ and which is also associated with cognitive flexibility.^51^ Sun et al. concurrently observed upregulation of inositol polyphosphate‐5‐phosphatase D (*INPP5D*) in cECs, as did Yang et al. (Figure 4D) and Bryant et al. across all ECs (Figure 4A). *INPP5D* is another gene also thought to mediate the microglial inflammatory response and is associated with both amyloid and tau pathology.^52^, ^53^, ^54^ Together with the presence of other immune‐related DEGs in cECs such as HLA class II histocompatibility antigen (*HLA‐DRB1*),^55^ altered expression of these genes may suggest roles for cECs in the neuroinflammatory aspects of disease progression, as opposed to solely microglia, the cell type on which efforts to understand the roles of these genes have been primarily concentrated. However, differential expression may also be related to more vascular aspects of disease pathology, such as *INPP5D*, which has also been found to be associated with cerebral blood flow dynamics in AD.^56^

**FIGURE 5:**
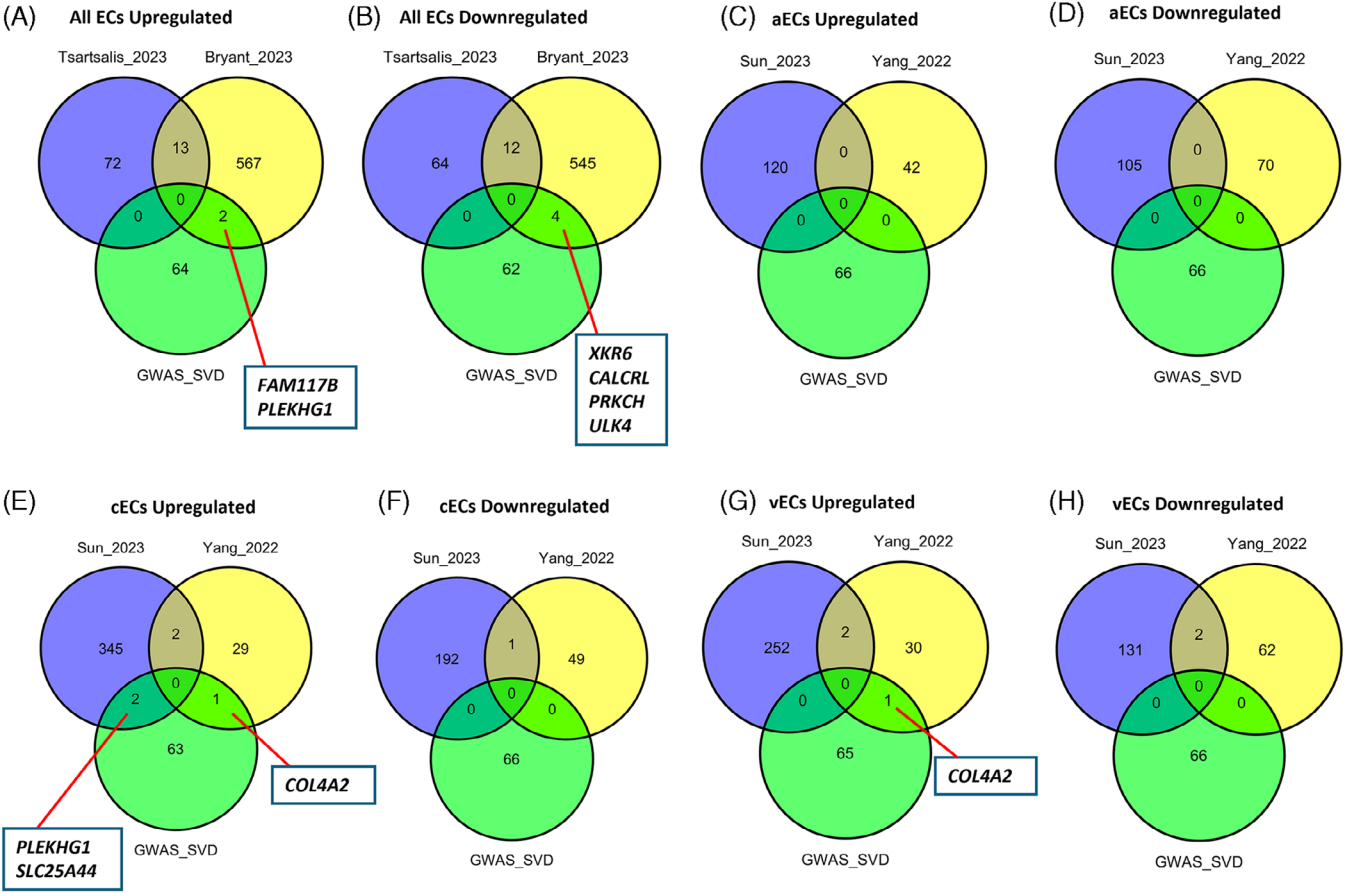
Comparison of AD studies’ EC DEGs with SVD WMH GWAS risk genes (obtained from Bhagat et al.). (A, B) Comparison for universal EC populations for aECs (C, D), for cECs (E, F), and for vECs (G, H). GWAS gene list obtained from^17^. AD, Alzheimer's disease; aECs, arterial ECs; cECs, capillary ECs; DEGs, differentially expressed genes; EC, endothelial cell; GWAS, genome‐wide association study; SVD, small vessel disease; vECs, venous ECs; WMH, white matter hyperintensity.

**FIGURE 6:**
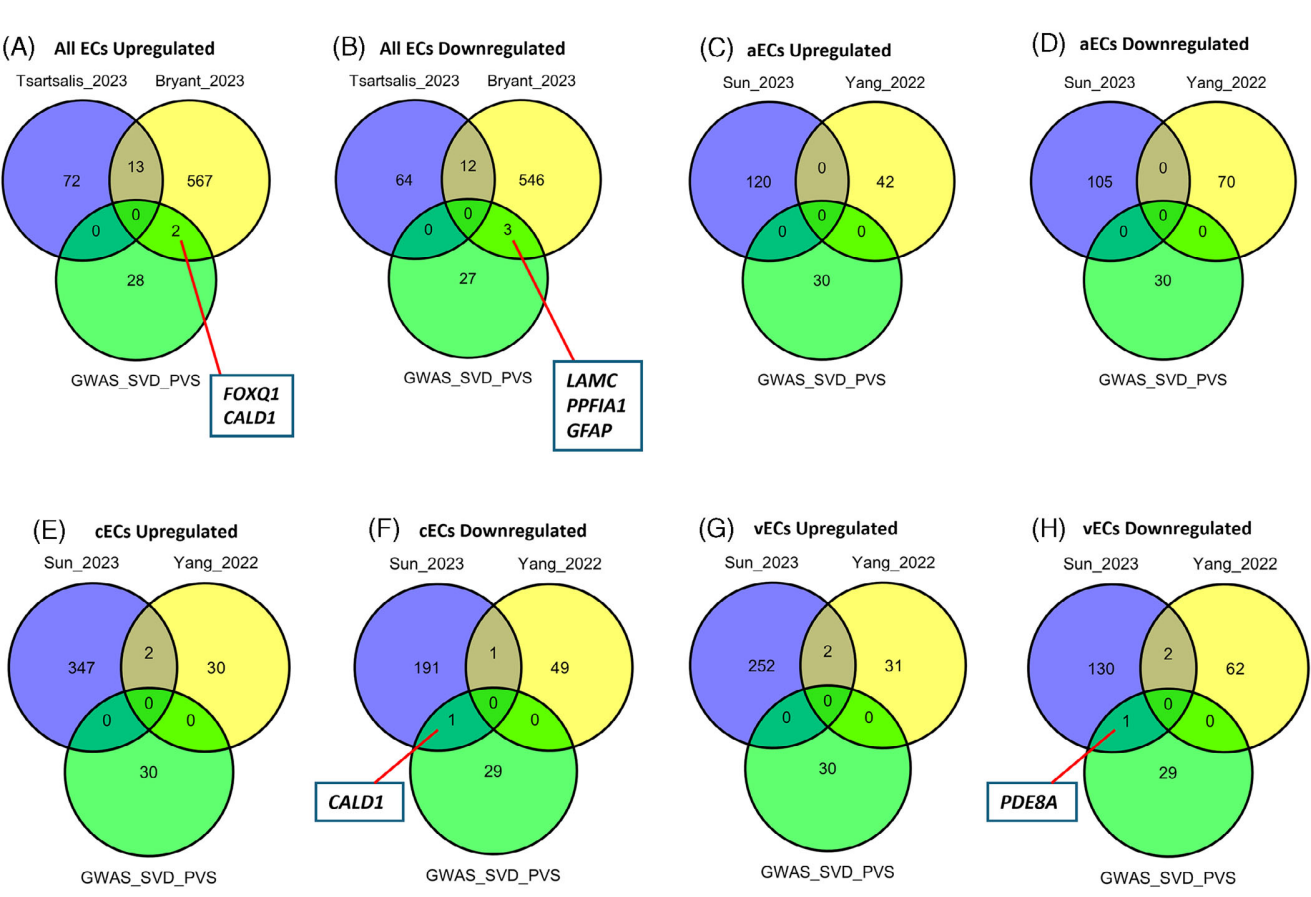
Comparison of AD studies’ EC DEGs with SVD PVS GWAS risk genes (obtained from Duperron et al.). (A, B) Comparison for universal EC populations for arterial aECs (C, D), for capillary cECs (E, F), and for venous vECs (G, H). GWAS gene list obtained from^18^. AD, Alzheimer's disease; aECs, arterial ECs; cECs, capillary ECs; DEGs, differentially expressed genes; EC, endothelial cell; GWAS, genome‐wide association study; PVS, perivascular space; SVD, small vessel disease; vECs, venous ECs.

Indeed, other evidence for associations with vascular abnormalities in AD may be inferred from examining whether these DEGs are associated with genetic risk for cerebral SVD, a common vascular comorbidity in patients with AD that has been proposed to drive AD pathology itself.^57^ We, therefore, explored whether any EC DEGs (universal ECs Figures 5 and 6A, B; aECs Figures 5 and 6C, D; cECs Figures 5 and 6E, F; vECs Figures 5 and 6G, H) in AD studies were associated with genetic risk for SVD white matter hyperintensity (WMH) (Figure 5) and perivascular space (PVS) burden (Figure 6), both of which are defining characteristics of SVD pathology that are also prevalent in patients with AD.^58^, ^59^ Several DEGs in AD postmortem tissue were found to be associated with SVD WMH genetic risk, including *COL4A2*, which was upregulated in both cECs (Figure 5E) and vECs (Figure 5G) in the Yang et al. study, and which encodes collagen at the basement membrane^60^; and *PLEKHG1*, encoding a Rho guanine exchange factor pleckstrin homology and RhoGEF domain containing G1,^61^ which was upregulated in ECs in the Bryant et al. study and in cECs in the Yang et al. study (Figure 5A,E). Several genes also appeared to be associated with PVS genetic risk, most notably *CALD1*, which was differentially expressed in ECs in the Bryant et al. study and in cECs in the Sun et al. study (Figure 6A,F). This gene encodes a cytoskeletal protein caldesmon 1,^62^ which may pertain to potential changes in EC adhesion in AD as discussed earlier.

**FIGURE 7:**
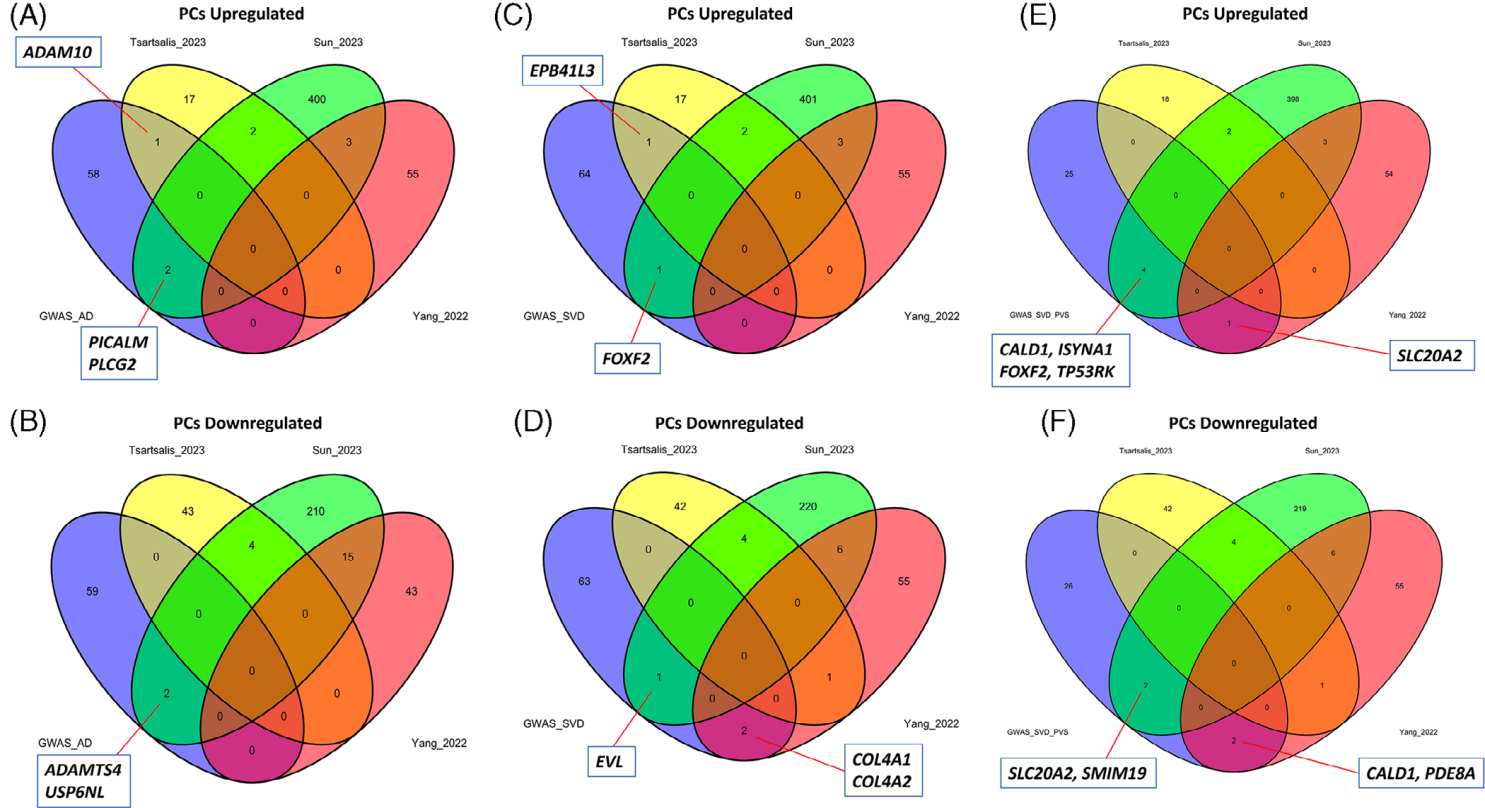
Comparison of AD studies’ pericyte DEGs with AD and SVD GWAS risk genes. (A, B) Venn diagrams showing overlap for upregulated (A) and downregulated (B) PC genes using the AD GWAS risk genes obtained from Tsartsalis et al.^13^ (C, D) Venn diagrams showing overlap for upregulated (C) and downregulated (D) PC genes using the SVD WMH GWAS risk genes obtained from Bhagat et al.^17^ (E, F) Venn diagrams showing overlap for upregulated (E) and downregulated (F) PC genes using the SVD PVS GWAS risk genes obtained from Duperron et al.^18^ AD, Alzheimer's disease; DEGs, differentially expressed genes; GWAS, genome‐wide association study; PC, pericyte; PVS, perivascular space; SVD, small vessel disease; WMH, white matter hyperintensity.

Our meta‐analysis also found several DEGs in PCs across studies that were associated with either AD or SVD WMH/PVS genetic risks (Figure 7). As for ECs, *PICALM* and *ADAM10*, a protease and regulator of NOTCH/EGFR signaling and amyloid precursor protein processing,^63^, ^64^ have been found to be upregulated in PCs in AD postmortem tissue (Figure 7A). Altered expression of genes associated with Aβ pathology were also noted in PCs, including *PLCG2* (Figure 7A) and *ADAMTS4* (Figure 7B).^65^, ^66^ As for ECs, the SVD WMH risk gene *COL4A2* has also been found to be differentially expressed in PCs, with the Yang et al. study identifying both *COL4A2* and *COL4A1* to be downregulated in AD PCs (Figure 7D). *FOXF2*, a risk gene encoding a forkhead‐box transcription factor associated with both SVD WMH and PVS, was also found to be upregulated in AD PCs in the Sun et al. study (Figure 7C,E), although it is plausible that this altered expression may be neuroprotective given that loss of *Foxf2* expression in adult mice promotes BBB breakdown.^67^ Similarly to ECs, *CALD1* was identified as a DEG in PCs in both the Sun et al. (Figure 7E) and Yang et al. (Figure 7F) studies. Both of these studies also found a PVS risk gene, the solute‐carrier family member and phosphate transporter *SLC20A2*, to be differentially expressed in PCs (Figure 7E,F). This is a gene of particular interest given that loss‐of‐function mutations of *SLC20A2* have been identified previously in patients with early‐onset AD and other neurodegenerative disorders associated with brain calcifications.^68^, ^69^ Solute‐like carrier family members are also known to be involved in blood–cerebrospinal fluid barrier maintenance,^70^ impairment of which due to altered expression of genes such as *SLC20A2* may lead to fluid accumulation in the PVS. Altogether, this analysis, therefore, provides evidence that certain AD/SVD genetic risk‐associated genes are also differentially expressed in vascular cells in AD pathology.

## 4 DISCUSSION

It is becoming increasingly clear from the ever‐expanding body of literature that changes to vascular cell molecular signatures are a key characteristic of the pathogenesis of neurodegenerative disorders such as AD, in addition to other glial cells such as microglia, which have garnered comparatively much more attention in recent years. Our analysis has identified several genes and pathways of interest that are found to be altered in human vascular cells for multiple pathologies, with several in AD tissue linked to genetic risk for AD and SVD pathologies. The role of these genes and their encoding proteins in the vascular etiology of these diseases thus warrants further investigation.

Examining the role of vascular transcriptomics in disease progression, however, has presented unique challenges to researchers, which have only just begun to be addressed in recent years. Notably, previous snRNA‐seq studies have typically captured very low yields of both endothelial and mural nuclei, which are often depleted during sample preparation for unknown reasons.^11^ Recent studies, however, have successfully developed, a‐nuclei enrichment protocols that have overcome this issue.^11^, ^13^ Indeed, Yang et al. were able to successfully capture distinct subtypes of ECs along the arteriovenous axis, which our analysis here suggests exhibit unique transcriptomic changes. Future work investigating the roles of these subsets, as well as those of mural cells such as PCs, will therefore likely need to continue to adopt such protocols. Zonal alterations may also be elucidated through other spatially focused transcriptomic technologies, which are a more recent and continuously developing addition to the omics field, in tandem with approaches such as snRNA‐seq. Spatial transcriptomics will also likely be needed to further and more directly understand how pathological hallmarks in these disorders with known impacts on the brain vasculature such as Aβ, influence vascular transcriptomes, as has been shown for other glial cells such as microglia and astrocytes using such an approach,^71^, ^72^ and to which many of the genes identified in our analysis appear to be associated.

Another fundamental question that has not yet been addressed in these diseases is how vascular transcriptomes are altered temporally across the trajectory of these diseases, which could shed light on which genes and how changes in their expression influence disease progression. Certain genes may that show greater changes at earlier stages of disease progression include apolipoprotein E (*APOE*), which has been shown recently to have enriched expression in certain vascular cell types,^11^, ^13^ with the *APOE4* allele also found to exacerbate aging and BBB breakdown.^6^ However, temporal changes have not been investigated in the literature we have examined here, which have all employed postmortem tissue to assess vascular alterations. It is therefore likely that omics approaches will need to be employed in other systems such as in vivo or stem‐cell–derived models to uncover potential longitudinal transcriptomic alterations. Such models will also be important for dissecting the functional impacts of individual genes in vascular cells during disease progression. Indeed, many of the genes identified as DEGs such as *INPP5D* across studies have been studied primarily in the context of microglia, with limited knowledge concerning their roles in vascular cells, and may be addressed in the future through the development of conditional vascular cell knock in/knockout models.

Genes that may be prioritized for further exploration with the development of such models include those associated with genetic risk, such as *INPP5D* for AD. However, much work also still needs to be done to advance our understanding of how genetic risk mediates gene expression in vascular cells. Although significant progress has been made through GWAS in identifying risk loci associated with diseases such as AD, several challenges remain in directly discerning the relevance of these findings to aspects of disease such as vascular pathology. Notably, these studies do not capture information regarding the impact of variants on specific cell types, and have found that the majority of these variants are located in non‐coding regions of the genome, such as in promoters and enhancers.^73^ Enrichment of AD risk variants specifically in ECs was recently implicated by Tsartsalis et al.^13^ through MAGMA analysis. However, whether any particular zonal EC subtype was enriched for risk loci was not investigated and should be explored in future omics‐focused studies. The impact of non‐coding variants on gene expression may also be determined in the future through other genomic approaches such as expression quantitative‐loci mapping and chromatin accessibility profiling specifically on vascular cells. Indeed, such approaches have successfully mapped risk variants to gene regulatory elements in microglia for AD.^74^, ^75^

A limitation of this meta‐analysis is that studies inevitably vary in their defining criteria for a gene to be considered as a “DEG,” such as differing log‐fold‐change threshold and *p*‐values. Although standardization of DEGs is inevitably challenging due to the differing analysis pipelines and nature of samples employed for sequencing across studies, the publication of more vascular transcriptomic studies will hopefully further narrow down candidate genes of interest. Nonetheless, the work of the studies analyzed here demonstrates the enormous progress made so far in advancing our understanding the role of the brain vasculature in neurodegenerative diseases, which in tandem with other future studies, will feed into the development of vascular‐targeted therapeutics.

## CONFLICT OF INTEREST STATEMENT

All datasets used in this article are publicly available in the referenced repositories. Further information will be readily available upon request. The authors declare no conflicts of interest. Author disclosures are available in the supporting information.

## CONSENT STATEMENT

Consent was not necessary because this study uses online public resources.

## Supporting information



Supporting Information

Supporting Information

## Data Availability

All data sets used in this study are publicly available and can be accessed via the referenced repositories. Further intermediate data and codes generated are available from the corresponding author upon request.

## References

[1] Wälchli T , Bisschop J , Carmeliet P , et al. Shaping the brain vasculature in development and disease in the single‐cell era. Nat Rev Neurosci. 2023;24:271‐298. doi:10.1038/s41583-023-00684-y 36941369 PMC10026800

[2] Sweeney MD , Sagare AP , Zlokovic BV . Blood‐brain barrier breakdown in Alzheimer disease and other neurodegenerative disorders. Nat Rev Neurol. 2018;14:133‐150. doi:10.1038/nrneurol.2017.188 29377008 PMC5829048

[3] Snyder HM , Corriveau RA , Craft S , et al. Vascular contributions to cognitive impairment and dementia including Alzheimer's disease. Alzheimers Dement J Alzheimers Assoc. 2015;11:710‐717. doi:10.1016/j.jalz.2014.10.008 PMC4731036 25510382

[4] Sweeney MD , Montagne A , Sagare AP , et al. Vascular dysfunction—the disregarded partner of Alzheimer's disease. Alzheimers Dement J Alzheimers Assoc. 2019;15:158‐167. doi:10.1016/j.jalz.2018.07.222 PMC6338083 30642436

[5] Chan ST , Mercaldo ND , Kwong KK , Hersch SM , Rosas HD . Impaired cerebrovascular reactivity in Huntington's disease. Front Physiol. 2021;12:663898. doi:10.3389/fphys.2021.663898 34366879 PMC8334185

[6] Montagne A , Nation DA , Sagare AP , et al. APOE4 leads to blood‐brain barrier dysfunction predicting cognitive decline. Nature. 2020;581:71‐76. doi:10.1038/s41586-020-2247-3 32376954 PMC7250000

[7] Nation DA , Sweeney MD , Montagne A , et al. Blood‐brain barrier breakdown is an early biomarker of human cognitive dysfunction. Nat Med. 2019;25:270‐276. doi:10.1038/s41591-018-0297-y 30643288 PMC6367058

[8] Khan F , Qiu H . Amyloid‐β: a potential mediator of aging‐related vascular pathologies. Vascul Pharmacol. 2023;152:107213. doi:10.1016/j.vph.2023.107213 37625763 PMC11793904

[9] Crouch EE , Joseph T , Marsan E , Huang EJ . Disentangling brain vasculature in neurogenesis and neurodegeneration using single‐cell transcriptomics. Trends Neurosci. 2023;46:551‐565. doi:10.1016/j.tins.2023.04.007 37210315 PMC10560453

[10] Vanlandewijck M , He L , Mäe MA , et al. A molecular atlas of cell types and zonation in the brain vasculature. Nature. 2018;554:475‐480. doi:10.1038/nature25739 29443965

[11] Yang AC , Vest RT , Kern F , et al. A human brain vascular atlas reveals diverse mediators of Alzheimer's risk. Nature. 2022;603:885‐892. doi:10.1038/s41586-021-04369-3 35165441 PMC9635042

[12] Sun N , Akay LA , Murdock MH , et al. Single‐nucleus multiregion transcriptomic analysis of brain vasculature in Alzheimer's disease. Nat Neurosci. 2023;26:970‐982. doi:10.1038/s41593-023-01334-3 37264161 PMC10464935

[13] Tsartsalis S , Sleven H , Fancy N , et al. A single nuclear transcriptomic characterisation of mechanisms responsible for impaired angiogenesis and blood‐brain barrier function in Alzheimer's disease. Nat Commun. 2024;15:2243. doi:10.1038/s41467-024-46630-z 38472200 PMC10933340

[14] Bryant A , Li Z , Jayakumar R , et al. Endothelial cells are heterogeneous in different brain regions and are dramatically altered in Alzheimer's disease. J Neurosci Off J Soc Neurosci. 2023;43:4541‐4557. doi:10.1523/JNEUROSCI.0237-23.2023 PMC10278684 37208174

[15] Garcia FJ , Sun N , Lee H , et al. Single‐cell dissection of the human brain vasculature. Nature. 2022;603:893‐899. doi:10.1038/s41586-022-04521-7 35158371 PMC9680899

[16] Winkler EA , Kim CN , Ross JM , et al. A single‐cell atlas of the normal and malformed human brain vasculature. Science. 2022;375:eabi7377. doi:10.1126/science.abi7377 35084939 PMC8995178

[17] Bhagat R , Marini S , Romero JR . Genetic considerations in cerebral small vessel diseases. Front Neurol. 2023;14:1080168. doi:10.3389/fneur.2023.1080168 37168667 PMC10164974

[18] Duperron M‐G , Knol MJ , Le Grand Q , et al. Genomics of perivascular space burden unravels early mechanisms of cerebral small vessel disease. Nat Med. 2023;29:950‐962. doi:10.1038/s41591-023-02268-w 37069360 PMC10115645

[19] Yu G , Wang L‐G , Han Y , He Q‐Y . clusterProfiler: an R package for comparing biological themes among gene clusters. Omics J Integr Biol. 2012;16:284‐287. doi:10.1089/omi.2011.0118 PMC3339379 22455463

[20] Szklarczyk D , Kirsch R , Koutrouli M , et al. The STRING database in 2023: protein‐protein association networks and functional enrichment analyses for any sequenced genome of interest. Nucleic Acids Res. 2023;51:D638‐646. doi:10.1093/nar/gkac1000 36370105 PMC9825434

[21] Li J , Li L , Cai S , Song K , Hu S . Identification of novel risk genes for Alzheimer's disease by integrating genetics from hippocampus. Sci Rep. 2024;14:27484. doi:10.1038/s41598-024-78181-0 39523385 PMC11551212

[22] Shannon P , Markiel A , Ozier O , et al. Cytoscape: a software environment for integrated models of biomolecular interaction networks. Genome Res. 2003;13:2498‐2504. doi:10.1101/gr.1239303 14597658 PMC403769

[23] Hu C , Yang J , Qi Z , et al. Heat shock proteins: biological functions, pathological roles, and therapeutic opportunities. MedComm. 2022;3:e161. doi:10.1002/mco2.161 35928554 PMC9345296

[24] Mehla J , Singh I , Diwan D , et al. STAT3 inhibitor mitigates cerebral amyloid angiopathy and parenchymal amyloid plaques while improving cognitive functions and brain networks. Acta Neuropathol Commun. 2021;9:193. doi:10.1186/s40478-021-01293-5 34911575 PMC8672532

[25] Korte N , Nortley R , Attwell D . Cerebral blood flow decrease as an early pathological mechanism in Alzheimer's disease. Acta Neuropathol (Berl). 2020;140:793‐810. doi:10.1007/s00401-020-02215-w 32865691 PMC7666276

[26] Rocha NP , Charron O , Colpo GD , et al. Cerebral blood flow is associated with markers of neurodegeneration in Huntington's disease. Parkinsonism Relat Disord. 2022;102:79‐85. doi:10.1016/j.parkreldis.2022.07.024 35973322

[27] March‐Diaz R , Lara‐Ureña N , Romero‐Molina C , et al. Hypoxia compromises the mitochondrial metabolism of Alzheimer's disease microglia via HIF1. Nat Aging. 2021;1:385‐399. doi:10.1038/s43587-021-00054-2 37117599

[28] Schulz JA , Hartz AMS , Bauer B . ABCB1 and ABCG2 regulation at the blood‐brain barrier: potential new targets to improve brain drug delivery. Pharmacol Rev. 2023;75:815‐853. doi:10.1124/pharmrev.120.000025 36973040 PMC10441638

[29] Nguyen YTK , Ha HTT , Nguyen TH , Nguyen LN . The role of SLC transporters for brain health and disease. Cell Mol Life Sci CMLS. 2021;79:20. doi:10.1007/s00018-021-04074-4 34971415 PMC11071821

[30] Drecourt A , Babdor J , Dussiot M , et al. Impaired transferrin receptor palmitoylation and recycling in neurodegeneration with brain iron accumulation. Am J Hum Genet. 2018;102:266‐277. doi:10.1016/j.ajhg.2018.01.003 29395073 PMC5985451

[31] Hossen F , Geng X , Sun GY , Yao X , Lee JC . Oligomeric Amyloid‐β and Tau Alter Cell Adhesion Properties and Induce Inflammatory Responses in Cerebral Endothelial Cells Through the RhoA/ROCK Pathway. Mol Neurobiol. 2024;61:8759‐8776. doi:10.1007/s12035-024-04138-z 38561558 PMC11445398

[32] Nguyen LK , Kholodenko BN , von Kriegsheim A . Rac1 and RhoA: networks, loops and bistability. Small GTPases. 2018;9:316‐321. doi:10.1080/21541248.2016.1224399 27533896 PMC5997137

[33] Melchionna R , Trono P , Tocci A , Nisticò P . Actin cytoskeleton and regulation of TGFβ signaling: exploring their links. Biomolecules. 2021;11:336. doi:10.3390/biom11020336 33672325 PMC7926735

[34] van Splunder H , Villacampa P , Martínez‐Romero A , Graupera M . Pericytes in the disease spotlight. Trends Cell Biol. 2024;34:58‐71. doi:10.1016/j.tcb.2023.06.001 37474376 PMC10777571

[35] Kapoor M , Chinnathambi S . TGF‐β1 signalling in Alzheimer's pathology and cytoskeletal reorganization: a specialized Tau perspective. J Neuroinflammation. 2023;20:72. doi:10.1186/s12974-023-02751-8 36915196 PMC10012507

[36] Medina‐Flores F , Hurtado‐Alvarado G , Deli MA , Gómez‐González B . The active role of pericytes during neuroinflammation in the adult brain. Cell Mol Neurobiol. 2023;43:525‐541. doi:10.1007/s10571-022-01208-5 35195811 PMC11415175

[37] Rustenhoven J , Aalderink M , Scotter EL , et al. TGF‐beta1 regulates human brain pericyte inflammatory processes involved in neurovasculature function. J Neuroinflammation. 2016;13:37. doi:10.1186/s12974-016-0503-0 26867675 PMC4751726

[38] Muraoka S , Jedrychowski MP , Yanamandra K , Ikezu S , Gygi SP , Ikezu T . Proteomic profiling of extracellular vesicles derived from cerebrospinal fluid of Alzheimer's disease Patients: a pilot study. Cells. 2020;9:1959. doi:10.3390/cells9091959 32854315 PMC7565882

[39] Wang L , Chiang H‐C , Wu W , et al. Epidermal growth factor receptor is a preferred target for treating amyloid‐β‐induced memory loss. Proc Natl Acad Sci USA. 2012;109:16743‐16748. doi:10.1073/pnas.1208011109 23019586 PMC3478595

[40] Chen Y‐J , Hsu C‐C , Shiao Y‐J , Wang H‐T , Lo Y‐L , Lin AMY . Anti‐inflammatory effect of afatinib (an EGFR‐TKI) on OGD‐induced neuroinflammation. Sci Rep. 2019;9:2516. doi:10.1038/s41598-019-38676-7 30792526 PMC6385176

[41] Montagne A , Barnes SR , Sweeney MD , et al. Blood‐brain barrier breakdown in the aging human hippocampus. Neuron. 2015;85:296‐302. doi:10.1016/j.neuron.2014.12.032 25611508 PMC4350773

[42] Smyth LCD , Highet B , Jansson D , et al. Characterisation of PDGF‐BB:PDGFRβ signalling pathways in human brain pericytes: evidence of disruption in Alzheimer's disease. Commun Biol. 2022;5:235. doi:10.1038/s42003-022-03180-8 35301433 PMC8931009

[43] Alam R , Mrad Y , Hammoud H , et al. New insights into the role of fibroblast growth factors in Alzheimer's disease. Mol Biol Rep. 2022;49:1413‐1427. doi:10.1007/s11033-021-06890-0 34731369

[44] Duan L , Zhang X‐D , Miao W‐Y , et al. PDGFRβ cells rapidly relay inflammatory signal from the circulatory system to neurons via chemokine CCL_2_ . Neuron. 2018;100:183‐200.e8. doi:10.1016/j.neuron.2018.08.030 30269986

[45] Ricciardelli AR , Robledo A , Fish JE , Kan PT , Harris TH , Wythe JD . The role and therapeutic implications of inflammation in the pathogenesis of brain arteriovenous malformations. Biomedicines. 2023;11:2876. doi:10.3390/biomedicines11112876 38001877 PMC10669898

[46] Saba J , Couselo FL , Bruno J , et al. Neuroinflammation in Huntington's disease: a starring role for astrocyte and microglia. Curr Neuropharmacol. 2022;20:1116‐1143. doi:10.2174/1570159X19666211201094608 34852742 PMC9886821

[47] Leng F , Edison P . Neuroinflammation and microglial activation in Alzheimer disease: where do we go from here? Nat Rev Neurol. 2021;17:157‐172. doi:10.1038/s41582-020-00435-y 33318676

[48] Ando K , Nagaraj S , Küçükali F , et al. PICALM and Alzheimer's disease: an update and perspectives. Cells. 2022;11:3994. doi:10.3390/cells11243994 36552756 PMC9776874

[49] Zhao Z , Sagare AP , Ma Q , et al. Central role for PICALM in amyloid‐β blood‐brain barrier transcytosis and clearance. Nat Neurosci. 2015;18:978‐987. doi:10.1038/nn.4025 26005850 PMC4482781

[50] Deczkowska A , Matcovitch‐Natan O , Tsitsou‐Kampeli A , et al. Mef2C restrains microglial inflammatory response and is lost in brain ageing in an IFN‐I‐dependent manner. Nat Commun. 2017;8:717. doi:10.1038/s41467-017-00769-0 28959042 PMC5620041

[51] Barker SJ , Raju RM , Milman NEP , et al. MEF2 is a key regulator of cognitive potential and confers resilience to neurodegeneration. Sci Transl Med. 2021;13:eabd7695. doi:10.1126/scitranslmed.abd7695 34731014 PMC9258338

[52] Chou V , Pearse RV , Aylward AJ , et al. INPP5D regulates inflammasome activation in human microglia. Nat Commun. 2023;14:7552. doi:10.1038/s41467-023-42819-w 38016942 PMC10684891

[53] Lin PB‐C , Tsai AP‐Y , Soni D , et al. INPP5D deficiency attenuates amyloid pathology in a mouse model of Alzheimer's disease. Alzheimers Dement J Alzheimers Assoc. 2023;19:2528‐2537. doi:10.1002/alz.12849 36524682

[54] Soni DM , Lin PB‐C , Lee‐Gosselin A , et al. Inpp5d haplodeficiency alleviates tau pathology in the PS19 mouse model of Tauopathy. Alzheimers Dement J Alzheimers Assoc. 2024;20:4985‐4998. doi:10.1002/alz.14078 PMC11247686 38923171

[55] Liu B , Shao Y , Fu R . Current research status of HLA in immune‐related diseases. Immun Inflamm Dis. 2021;9:340‐350. doi:10.1002/iid3.416 33657268 PMC8127548

[56] Yao X , Risacher SL , Nho K , et al. Targeted genetic analysis of cerebral blood flow imaging phenotypes implicates the INPP5D gene. Neurobiol Aging. 2019;81:213‐221. doi:10.1016/j.neurobiolaging.2019.06.003 31319229 PMC6732252

[57] Paolini Paoletti F , Simoni S , Parnetti L , Gaetani L . The contribution of small vessel disease to neurodegeneration: focus on Alzheimer's disease, Parkinson's disease and multiple sclerosis. Int J Mol Sci. 2021;22:4958. doi:10.3390/ijms22094958 34066951 PMC8125719

[58] Garnier‐Crussard A , Cotton F , Krolak‐Salmon P , Chételat G . White matter hyperintensities in Alzheimer's disease: beyond vascular contribution. Alzheimers Dement J Alzheimers Assoc. 2023;19:3738‐3748. doi:10.1002/alz.13057 37027506

[59] Lynch M , Pham W , Sinclair B , O'Brien TJ , Law M , Vivash L . Perivascular spaces as a potential biomarker of Alzheimer's disease. Front Neurosci. 2022;16:1021131. doi:10.3389/fnins.2022.1021131 36330347 PMC9623161

[60] Meuwissen MEC , Halley DJJ , Smit LS , et al. The expanding phenotype of COL4A1 and COL4A2 mutations: clinical data on 13 newly identified families and a review of the literature. Genet Med Off J Am Coll Med Genet. 2015;17:843‐853. doi:10.1038/gim.2014.210 25719457

[61] Traylor M , Tozer DJ , Croall ID , et al. Genetic variation in PLEKHG1 is associated with white matter hyperintensities (n = 11,226). Neurology. 2019;92:e749‐757. doi:10.1212/WNL.0000000000006952 30659137 PMC6396967

[62] Mayanagi T , Sobue K . Diversification of caldesmon‐linked actin cytoskeleton in cell motility. Cell Adhes Migr. 2011;5:150‐159. doi:10.4161/cam.5.2.14398 PMC3084981 21350330

[63] Liao S , Lin Y , Liu L , et al. ADAM10‐a “multitasker” in sepsis: focus on its posttranslational target. Inflamm Res Off J Eur Histamine Res Soc Al. 2023;72:395‐423. doi:10.1007/s00011-022-01673-0 PMC9789377 36565333

[64] Khezri MR , Mohebalizadeh M , Ghasemnejad‐Berenji M . Therapeutic potential of ADAM10 modulation in Alzheimer's disease: a review of the current evidence. Cell Commun Signal CCS. 2023;21:60. doi:10.1186/s12964-023-01072-w 36918870 PMC10012555

[65] Tsai AP , Dong C , Lin PB‐C , et al. PLCG2 is associated with the inflammatory response and is induced by amyloid plaques in Alzheimer's disease. Genome Med. 2022;14:17. doi:10.1186/s13073-022-01022-0 35180881 PMC8857783

[66] Matsuzaki M , Yokoyama M , Yoshizawa Y , et al. ADAMTS4 is involved in the production of the Alzheimer disease amyloid biomarker APP669‐711. Mol Psychiatry. 2023;28:1802‐1812. doi:10.1038/s41380-023-01946-y 36721026 PMC10208957

[67] Reyahi A , Nik AM , Ghiami M , et al. Foxf2 is required for brain pericyte differentiation and development and maintenance of the blood‐brain barrier. Dev Cell. 2015;34:19‐32. doi:10.1016/j.devcel.2015.05.008 26120030

[68] Wang C , Li Y , Shi L , et al. Mutations in SLC20A2 link familial idiopathic basal ganglia calcification with phosphate homeostasis. Nat Genet. 2012;44:254‐256. doi:10.1038/ng.1077 22327515

[69] Hebestreit S , Schwahn J , Sandikci V , et al. PSEN1/SLC20A2 double mutation causes early‐onset Alzheimer's disease and primary familial brain calcification co‐morbidity. Neurogenetics. 2023;24:209‐213. doi:10.1007/s10048-023-00723-x 37341843 PMC10319679

[70] Ho HTB , Dahlin A , Wang J . Expression profiling of solute carrier gene families at the blood‐CSF barrier. Front Pharmacol. 2012;3:154. doi:10.3389/fphar.2012.00154 22936914 PMC3426838

[71] Mallach A , Zielonka M , van Lieshout V , et al. Microglia‐astrocyte crosstalk in the amyloid plaque niche of an Alzheimer's disease mouse model, as revealed by spatial transcriptomics. Cell Rep. 2024;43:114216. doi:10.1016/j.celrep.2024.114216 38819990

[72] Chen W‐T , Lu A , Craessaerts K , et al. Spatial transcriptomics and in situ sequencing to study Alzheimer's disease. Cell. 2020;182:976‐991.e19. doi:10.1016/j.cell.2020.06.038 32702314

[73] Romero‐Molina C , Garretti F , Andrews SJ , Marcora E , Goate AM . Microglial efferocytosis: diving into the Alzheimer's disease gene pool. Neuron. 2022;110:3513‐3533. doi:10.1016/j.neuron.2022.10.015 36327897 PMC13175419

[74] Nott A , Holtman IR , Coufal NG , et al. Brain cell type‐specific enhancer‐promoter interactome maps and disease‐risk association. Science. 2019;366:1134‐1139. doi:10.1126/science.aay0793 31727856 PMC7028213

[75] Kosoy R , Fullard JF , Zeng B , et al. Genetics of the human microglia regulome refines Alzheimer's disease risk loci. Nat Genet. 2022;54:1145‐1154. doi:10.1038/s41588-022-01149-1 35931864 PMC9388367

