# Supplementary material for: Unraveling the transcriptomic landscape of brain vascular cells in dementia: A systematic review
Source: Alzheimers Dement. 2025 Jan 14;21(2):e14512. doi: 10.1002/alz.14512 (PMC11851133; doi:10.1002/alz.14512)
Supplement: Supplementary file 1 — Supporting Information [file ALZ-21-e14512-s002.docx]

**Supplementary Figures**


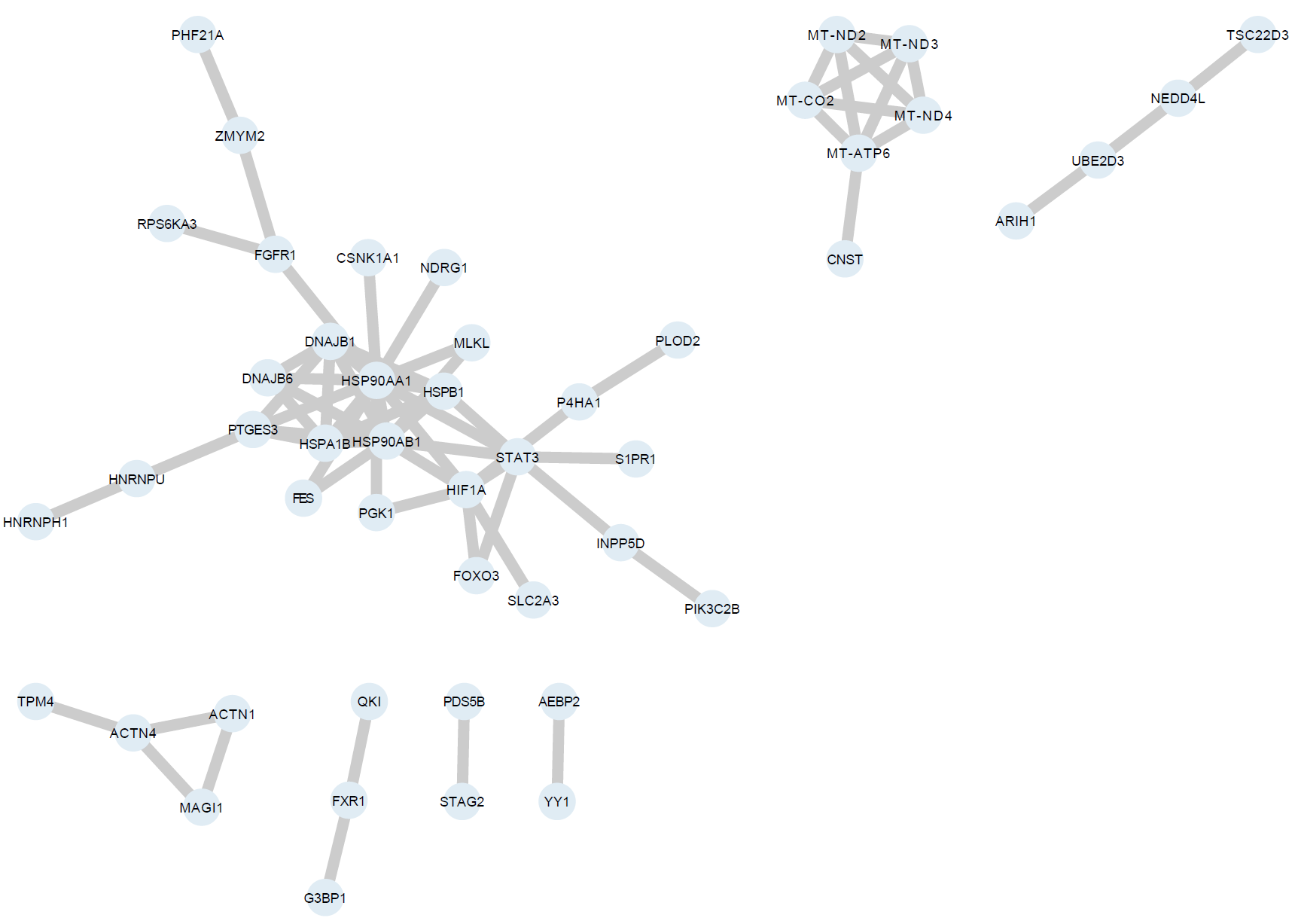
**Supplementary Figure 1. PPI network generated from STRING for shared upregulated endothelial cell genes between Garcia *et al.* (HD) and either Bryant *et al.*/Tsartsalis *et al.* (AD) studies.** Interactions displayed are those determined by STRING to be of high confidence.


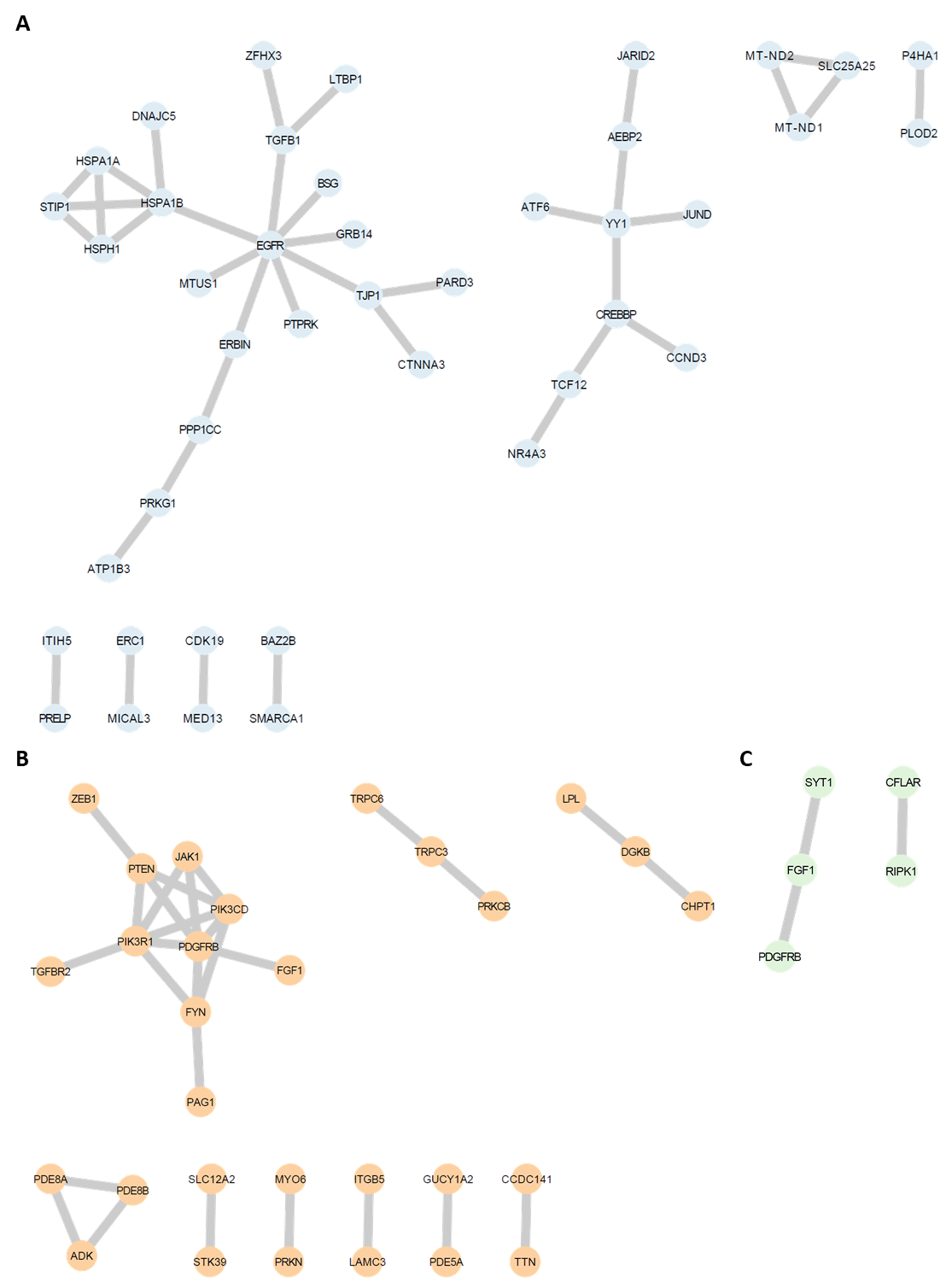


**Supplementary Figure 2. PPI network generated by STRING for shared upregulated and downregulated pericyte genes between Garcia *et al.* (HD), Winkler *et al.* (AVM) and Sun *et al.*/Yang *et al.*/Tsartsalis *et al.* (AD) studies.** (**A**) PPI network generated by STRING for shared upregulated pericyte genes between Garcia *et al.* (HD) and either one of Sun *et al.*/Yang *et al.*/Tsartsalis *et al.* (AD) studies. (**B**, **C**) PPI networks for shared downregulated genes between Garcia *et al.* (HD), and (**B**) Winkler *et al.* (AVM), and (**C**) Tsartsalis *et al.*/Sun *et al.*/ Yang *et al.* (AD). Interactions displayed are those determined by STRING to be of high confidence.

**Supplementary Tables**

**Supplementary Table 1A. Full search strategy.**

| 1. | exp Endothelium, Vascular/ or exp Endothelium/ or Endothelium.mp. |
| --- | --- |
| 2. | Microvasculature.mp. or exp Microvessels/ |
| 3. | exp Endothelial Cells/ or Vasculature.mp. or exp Blood Vessels/ |
| 4. | 1 or 2 or 3 |
| 5. | exp Sequence Analysis, RNA/ or exp Gene Expression Profiling/ or exp Single-Cell Analysis/ or single-cell RNA-sequencing.mp. or exp Transcriptome/ |
| 6. | single-nuclei RNA-sequencing.mp. or exp single-cell/ or single-cell.mp. or exp single-nuclei/ or single-nuclei.mp. |
| 7. | 5 or 6 |
| 8. | Neurodegeneration.mp. or exp Neurodegenerative Diseases/ |
| 9. | 4 and 7 and 8 |
| 10. | exp Brain/ or Brain.mp. |
| 11. | 9 and 10 |
| 12. | limit 11 to (humans and last 5 years) |

**Supplementary Table 1B. List of studies.**

|  | **Title** | **Year** | **Journal** | **Authors** | **Content** | **Log2FC threshold for DEG** | **Adjusted p-value threshold for DEG** | **Other threshold criteria** |
| --- | --- | --- | --- | --- | --- | --- | --- | --- |
| **1.** | A single nuclear transcriptomic characterisation of mechanisms responsible for impaired angiogenesis and blood-brain barrier function in Alzheimer’s disease | 2024 | Nature Communications | Tsartsalis *et al.* | human: AD, age-matched controls; EC and PC, no SMC | N/A | 0.1 | N/A |
| **2.** | Endothelial Cells Are Heterogeneous in Different Brain Regions and Are Dramatically Altered in Alzheimer's Disease | 2023 | Journal of Neuroscience | Bryant *et al.* | human: AD and age-matched controls; EC cells and their subtypes | ≥ +0.1 (upregulated) or ≤ -0.1 (downregulated) | 0.1 | Expressed in > 10% of nuclei |
| **3.** | Single-nucleus multiregion transcriptomic analysis of brain vasculature in Alzheimer’s disease | 2023 | Nature Neuroscience | Sun *et al.* | human: AD and age-matched controls; subtypes of EC, PC, SMC | N/A | N/A | MAST coefficient > +0.02 (upregulated) and < -0.02 (downregulated), and p-value < 0.01 |
| **4.** | Single-cell dissection of the human brain vasculature | 2022 | Nature | Garcia *et al.* | human: HD and age-matched controls; subtypes of EC, PC, SMC | ≥ +0.05 (upregulated) or ≤ -0.05 (downregulated) | 0.05 | N/A |
| **5.** | A human brain vascular atlas reveals diverse mediators of Alzheimer’s risk | 2022 | Nature | Yang *et al.* | human: age-matched controls and AD, subtypes of EC, PC, SMC | ≥ +0.3 (upregulated) or ≤ -0.3 (downregulated) | 0.1 | Expressed in > 10% of nuclei |
| **6.** | A single-cell atlas of the normal and malformed human brain vasculature | 2022 | Science | Winkler *et al.* | human: AVM and age-matched controls; EC, SMC subtypes but not PC | N/A | 0.05 | N/A |

**Supplementary Table 2A. Overlapping upregulated genes between Bryant, Garcia, and Tsartsalis studies for GO enrichment analysis.**

| **Genes** |  |  |  |  |  |
| --- | --- | --- | --- | --- | --- |
| HSPA1B | FXR1 | GPBP1 | TRIM26 | PTPN14 | RPS6KA5 |
| HSP90AA1 | FAM13A | SESTD1 | G3BP1 | INPP5D | GNA14 |
| DNAJB1 | STARD4 | ARL5B | CARHSP1 | TRIM56 | RTN1 |
| HSPB1 | TSC22D3 | ANKRD12 | FGFR1 | ELL2 | PTPRE |
| PTGES3 | DHFR | QKI | HNRNPH1 | RAPGEF5 | ATP11C |
| HSP90AB1 | PTMS | LDLR | TPM4 | KIF13A | ATP1B3 |
| P4HA1 | GBE1 | MLKL | CLEC1A | SIK2 | MAGI1 |
| MT-ND3 | NEDD4L | PLEKHG1 | INTS6 | SEMA6B | PHF21A |
| SLC38A2 | NAA16 | USP34 | ACTN4 | KAT6A | CEP112 |
| DNAJB6 | STAU2 | UBE2D3 | RAB21 | SYTL4 | RFX3 |
| FMNL2 | CALCOCO2 | NDRG1 | PDS5B | ARIH1 | FANCC |
| MT-ATP6 | PGK1 | CNST | RHOJ | ANKRD17 | AEBP2 |
| HNRNPU | MT-ND4 | RANGAP1 | SPEN | CSNK1A1 | PPP3CA |
| PLOD2 | MPDZ | UVRAG | ST3GAL6 | SMARCA2 | TTBK2 |
| CREM | YY1 | TMTC2 | FOXO3 | SORBS1 | TWSG1 |
| MT-CO2 | S1PR1 | STAT3 | HP1BP3 | CSGALNACT1 | ZMYM2 |
| MT-ND2 | SLCO4A1 | CRIM1 | DYSF | PIK3C2B | MECOM |
| RPS6KA3 | OSBPL1A | ST8SIA6 | MTCH1 | FES | ST7 |
| CLK1 | FOSL2 | MORF4L1 | SGPP2 | ACTN1 |  |
| CSMD1 | PELI1 | SLC2A3 | STAG2 | HIF1A |  |

**Supplementary Table 2B. Overlapping upregulated genes between Garcia and Winkler studies.**

| **Genes** |  |
| --- | --- |
| AGO2 | MAGI1 |
| ANO6 | MCTP1 |
| ASRGL1 | MYO1D |
| ATP1B3 | PLAUR |
| BACE2 | PTGES3 |
| CALCRL | PTPRE |
| CMIP | RALGAPA2 |
| CNST | SLC38A2 |
| DNAJB1 | SNTB2 |
| DOCK4 | SPSB1 |
| ECE1 | TGFBR3 |
| FAM107B | TLL1 |
| FRMD4B | TSHZ2 |
| HSPG2 | ZNF385D |
| IQCJ-SCHIP1 | ZNF608 |
| ITGA10 |  |
| ITGA2 |  |
| KCTD8 |  |
| LRRC1 |  |
| MACF1 |  |

**Supplementary Table 2C. Overlapping downregulated genes between Garcia and Winkler studies (subsequently used for GO enrichment analysis).**

| **Genes** |  |  |  |  |
| --- | --- | --- | --- | --- |
| HLA-C | ABCB1 | TMEM132C | ITFG1 | SEMA3G |
| RUNDC3B | SLC9A3R2 | FAXDC2 | XPR1 | CHKA |
| ABCG2 | ADIRF | NAALADL2 | SLC30A1 | SENP7 |
| IFI27 | IFI44L | CTNNB1 | MAP4 | TMEM50B |
| GPR85 | HERPUD1 | EPSTI1 | SLC16A14 | SLFN5 |
| LMO2 | PARD3B | JCAD | SH3BP4 | PLLP |
| B2M | BTNL9 | MTUS1 | ABCD3 | EPAS1 |
| ANXA3 | TFRC | CCSER2 | FKBP9 | KCTD3 |
| KLHL5 | PARP14 | PCMTD1 | CKB | DEGS2 |
| MFSD2A | USP54 | TBC1D4 | DCLK1 | FAM120B |
| SLC16A1 | GALNT15 | NR3C1 | LIMS2 | NTNG1 |
| THSD4 | C22orf34 | STXBP6 | SRGAP2C | MYEF2 |
| SNED1 | WSB1 | LAMA3 | CD34 | NPTN |
| SLC2A1 | IFNGR1 | ERG | HSPA12B | LIFR |
| HEG1 | CD46 | MX1 | NEK3 |  |
| PTPRB | MCC | GBP4 | TNRC6A |  |
| KLF2 | ANKRD29 | PHKB | PROM1 |  |
| HLF | SLC38A5 | TMCC3 | EPHA3 |  |
| ITGA6 | ITGA1 | PODXL | TIE1 |  |
| CAVIN2 | AIF1L | GTDC1 | JAG2 |  |

**Supplementary Table 2D. Overlapping downregulated genes between Bryant, Garcia and Tsartsalis studies for GO enrichment analysis.**

| **Genes** |  |  |  |  |  |  |  |
| --- | --- | --- | --- | --- | --- | --- | --- |
| USP25 | PREX2 | PRTG | APBB2 | MIA2 | ARMCX4 | PKD2 | RAPH1 |
| TMX3 | FAM135A | SORBS2 | FKBP9 | TDRD3 | VWA8 | GALNT17 | ADGRF5 |
| HSPA12B | PSMD1 | DMXL1 | SHPRH | SNED1 | ULK4 | CFH | DCLK1 |
| GALNT11 | NUDCD3 | ADCY4 | RPAP2 | CIRBP | SLFN12L | GPC5 | CHKA |
| CYYR1 | CLNS1A | FBXW11 | TTC17 | TGFBR2 | SCFD2 | HLCS | RAC1 |
| TCERG1 | ZZEF1 | GRB2 | ARHGAP12 | KLC1 | SH3D19 | RFTN1 | CLIC5 |
| RNF111 | DNAJC10 | IREB2 | KATNBL1 | COA1 | CEP192 | THSD4 | RANBP17 |
| FGD6 | KIAA1841 | ADAM15 | WDR43 | ZNF721 | MAPKAP1 | SUMF1 | PPP3CC |
| NBDY | DNAJC13 | ODF2L | TRMT11 | KANK3 | JAK2 | PALMD | TEK |
| CKAP5 | HEG1 | SPAAR | FBXO38 | MGLL | SHROOM4 | ETS1 | MAP4 |
| ANAPC10 | XPR1 | DCAF8 | IFNGR1 | ZDHHC21 | ABCD3 | JARID2 | NEK3 |
| ADK | KLHDC10 | RECK | EFR3B | AVL9 | ACSL5 | NAALADL2 | MX2 |
| TTC37 | EXOC1 | PUM3 | EPSTI1 | RUNDC3B | SMAD6 | MCC | TACC1 |
| U2SURP | CUL5 | SCAF8 | AGO4 | GPATCH8 | KIAA0319L | FLI1 | TLE4 |
| WDR20 | JCAD | TOX2 | LONP2 | SCMH1 | PARD3B | CP | SPTBN1 |
| MTM1 | TBC1D9B | TRPC1 | FAM107A | PHC2 | ZMAT1 | XAF1 | FBXW2 |
| STIM1 | CDC14A | PPIP5K2 | RBM26 | MYEF2 | BBS9 | USP54 | ZNF91 |
| WRN | SPTLC2 | TMCC3 | KLHL5 | SNRK | GALNT15 | RASAL2 | HABP4 |
| PER3 | APPL2 | SH3BP4 | SECISBP2 | MNAT1 | PAPSS2 | NOSTRIN |  |
| EMCN | LIMS2 | PPP1R21 | RBM25 | SBF2 | HGSNAT | CELF2 |  |

**Supplementary Table 3A. Overlapping upregulated pericyte genes for GO enrichment analysis from Garcia, Sun, Tsartsalis, and Yang studies.**

| **Genes** |  |  |  |  |  |
| --- | --- | --- | --- | --- | --- |
| PCDH9 | EEF1D | MKLN1 | SLC25A25 | COL27A1 | SPPL3 |
| UNC5C | EGFR | MT-ND1 | SLC38A2 | CREBRF | STAG1 |
| CTNNA3 | ERC1 | MT-ND2 | SLC6A12 | ESYT2 | TACC2 |
| POU6F2 | FAM13A | MTUS1 | SNX25 | GABARAPL1 | ZBTB20 |
| NEBL | FAM193A | MXI1 | SSTR2 | HECTD4 | ZFHX3 |
| MAST4 | GEM | NR4A3 | STIP1 | HIC1 | ERBIN |
| AEBP2 | GOLPH3 | NTRK3 | SYTL4 | HNRNPU | VPS13A |
| ATF6 | GRAMD2B | P4HA1 | TCF12 | ITIH5 |  |
| ATP1B3 | GRB14 | PDE1C | TGFB1 | JARID2 |  |
| BAZ2B | HELZ | PICALM | TJP1 | LPP |  |
| BSG | HSPA1A | PLOD2 | TSC22D3 | N4BP2L2 |  |
| CALD1 | HSPA1B | PPM1L | UBE2E1 | NR2C2 |  |
| CARHSP1 | HSPH1 | PPP1CC | USP15 | PARD3 |  |
| CCND3 | ISYNA1 | PRELP | YY1 | POLR2H |  |
| CDK19 | ITGA11 | PRKG1 | ZFP36L1 | RNF115 |  |
| CENPP | JUND | PTMS | ZIC1 | RNF152 |  |
| COL18A1 | KLF9 | PTPRK | ZNF407 | SASH1 |  |
| CREBBP | LTBP1 | RBM17 | APOLD1 | SMARCA1 |  |
| DNAJC5 | MED13 | RFX3 | BHLHE40 | SMG6 |  |
| DYM | MICAL3 | SCAPER | CDC42EP4 | SOX5 |  |

**Supplementary Table 3B. Overlapping downregulated pericyte genes for GO enrichment analysis from Garcia and Winkler studies.**

| **Genes** |  |  |  |  |  |
| --- | --- | --- | --- | --- | --- |
| CDH6 | FRMD3 | PID1 | DSE | SRSF5 | PIK3CD |
| PDGFRB | DCN | ARGLU1 | CPQ | TGFBR2 | PLA2R1 |
| RAPH1 | C2CD2 | CYSLTR2 | WLS | PPARGC1B | CNTN4 |
| SLC6A1 | MPPED2 | HIGD1B | TESC | SPECC1 | FEZ2 |
| PAG1 | RAPGEF5 | ACSS3 | PDE8A | MYO6 | TNFRSF21 |
| SLC38A11 | GRM8 | TRPC6 | PDZD2 | PRKCB | PER3 |
| SLC1A3 | P2RY14 | NHSL1 | KLHL23 | RPS6KA2 | PGRMC1 |
| PTN | GPC5 | PRKN | TRPC3 | LAMC3 | SMYD3 |
| PDE8B | MED12L | GRK3 | BBS9 | CHPT1 | ENDOD1 |
| GUCY1A2 | CD9 | GRM3 | TTN | JAK1 | LBH |
| PTEN | RERG | ZSWIM5 | EVL | ANO10 | PLAC9 |
| MYO1B | RGS5 | PDE5A | ZNF254 | ANKRD36 | MMP16 |
| SNRK | SNTB1 | SLC20A2 | LPL | ADAP2 | CCDC141 |
| CA2 | CIRBP | ZEB1 | PPFIBP1 | LIN7A | C20orf194 |
| FGF1 | AUTS2 | ADK | PLCL1 | ITM2C |  |
| GGT5 | PAPSS2 | PTPN9 | CTDSPL | LRMDA |  |
| SLC12A2 | ADGRF5 | ATP1A2 | DGKB | DAAM2 |  |
| NDUFA4L2 | SLC19A1 | SPTBN1 | PLXDC1 | PIK3R1 |  |
| CCDC3 | BLCAP | KANK3 | FYN | STK39 |  |
| ITGB5 | GRK5 | RFTN1 | MTSS1 | COBLL1 |  |

**Supplementary Table 3C. Overlapping downregulated pericyte genes for GO enrichment analysis from Garcia, Sun, Tsartsalis, and Yang studies.**

| **Genes** |  |  |
| --- | --- | --- |
| PDE8A | MPRIP | RIPK1 |
| CA2 | NFASC | TTC37 |
| REV3L | NKD1 | UGGT2 |
| SLC12A2 | PDGFRB | USP13 |
| CYSLTR2 | PLEKHG2 | MAP1B |
| COL4A2 | PPARGC1B | MAP4 |
| PCBP3 | RFTN1 | GRK5 |
| ACTN4 | SLC19A1 | LIN7A |
| ATP1A2 | SLC20A2 | RPS6KA2 |
| C2CD2 | SLC6A1 | TNS3 |
| CIRBP | SYT1 | NHSL1 |
| CTNNBL1 | TMEM168 | CFLAR |
| DGKB | TPM1 | SLC2A12 |
| DKK3 | CUL3 | HLCS |
| EVL | DDX5 |  |
| FGF1 | DLGAP4 |  |
| GRM3 | EDIL3 |  |
| GRM8 | ERCC8 |  |
| MLLT3 | GRK3 |  |
| MPPED2 | PLEKHG3 |  |
